# Bladder Leiomyoma with Synchronous Solitary Fibrous Tumor of the Pleura

**DOI:** 10.1155/2020/3717506

**Published:** 2020-02-25

**Authors:** Charalampos Mavridis, George Georgiadis, Eleni D. Lagoudaki, Iordanis Skamagkas, Ioannis Heretis, Anastasios V. Koutsopoulos, Charalampos Mamoulakis

**Affiliations:** ^1^Department of Urology, University General Hospital of Heraklion, University of Crete, Medical School, Heraklion, Crete, Greece; ^2^Department of Pathology, University General Hospital of Heraklion, University of Crete, Medical School, Heraklion, Crete, Greece

## Abstract

Bladder leiomyomas (BLs) are extremely rare benign tumors of mesenchymal origin. The exact pathophysiological mechanisms that lead to their appearance remain unclear including hormonal disorders, chromosomal abnormalities, and fetal remnants in the bladder. They usually remain asymptomatic for a long period of time. Solitary fibrous tumors (SFTs) are also rare neoplasms of mesenchymal origin with malignant potential usually affecting the pleura. The pathogenesis of SFTs remains unclear. We report the case of a 28-year-old male presenting with SFT of the pleura and synchronous BL. The patient presented with persistent cough as a sole symptom. Computed tomography (CT) of the thorax revealed a pleural mass, which was surgically removed and proved to be a SFT. At an early follow-up, abdominal CT scan revealed a bladder wall mass that proved to be a BL. This is the first report of BL with synchronous SFT of the pleura. Synchronous BLs and SFTs may be incidental, but the coexistence of two mesenchymal tumors at different sites, in a young patient, may raise the suspicion of a new clinical syndrome that warrants further investigation.

## 1. Introduction

Bladder leiomyomas (BLs) are very rare benign tumors of mesenchymal origin (<0.5%, <250 cases have been published) [1]. The exact pathophysiological mechanisms that lead to their appearance remain unclear. Several theories have been proposed for the interpretation of their development such as hormonal disorders, vestigial remnants in the bladder, perivascular inflammation-metaplasia of bladder structures, chromosomal abnormalities, and bladder wall infection or inflammation [2]. They are usually asymptomatic (80%) [1], manifesting rarely with hematuria and urge or obstructive urination depending on the localization (endovesical: 63%, extravesical: 30%, and intramural: 7%) [2, 3]. Solitary fibrous tumors (SFTs) are also very rare tumors of mesenchymal origin, usually affecting the pleura. Less than 800 cases have been published globally, accounting for 5% of chest tumors [4]. Extrapleural SFTs are commonly located at the visceral skull and the retroperitoneum [5]. The presence of SFTs in the genitourinary system is extremely rare (14 cases of prostate SFTs [6] and <50 cases of renal SFTs [7] have been published). The pathogenesis of SFTs has not been elucidated, and they usually remain asymptomatic [5]. The aim of this report is to present a unique case of pleural SFT with synchronous BL.

## 2. Case Presentation

**Figure 1:**
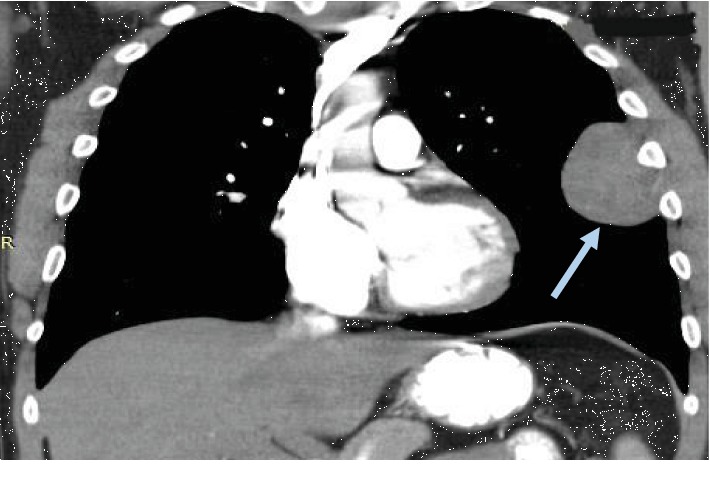
Solitary fibrous tumor of the left pleura (grey arrow). Computed tomography, thorax. Coronal view.

**Figure 2:**
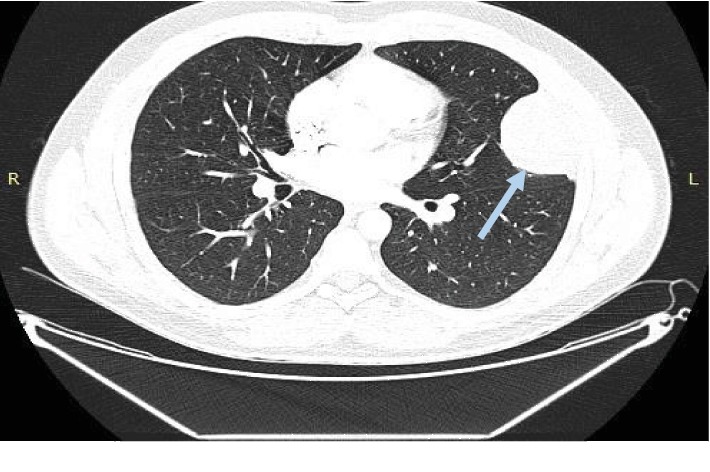
Solitary fibrous tumor of the left pleura (grey arrow). Computed tomography, thorax. Horizontal view.

**Figure 3:**
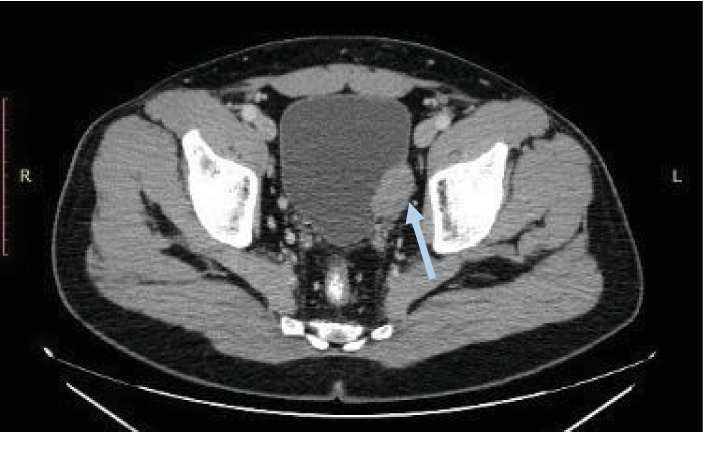
Bladder leiomyoma (grey arrow). Computed tomography, low abdomen. Horizontal view.

**Figure 4:**
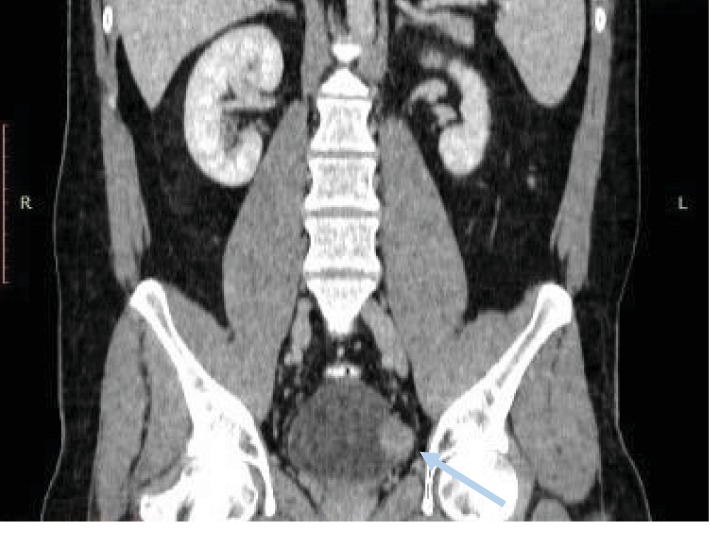
Bladder leiomyoma (grey arrow). Computed tomography, abdomen. Coronal view.

A 28-year-old male visited the outpatient pulmonary clinic of our hospital due to persistent cough during the last six months. Medical history and physical examination did not reveal any pathology. Computed tomography (CT) scan of the thorax showed a tumor (6.8 × 5.5 × 4.5 cm) of the left pleura (Figures 1 and 2). The mass was surgically removed and proved to be a pleural tumor with varying cellularity composed of uniform spindle cells with fibroblastic morphology and rare mitotic activity (0-1 MF/10 HPF) arranged in a “patternless pattern” or forming short ill-defined fascicles in an extensively collagenized and regionally myxoid stroma. Immunohistochemically, tumor cells expressed CD34, vimentin, and Bcl-2, showing no expression of smooth muscle actin (SMA), EMA, Cam 5.2, desmin, and calretinin. These findings were compatible with a SFT of the pleura [6]. At the time of diagnosis, imaging of the lower abdomen was not performed. During a routine follow-up at 6 months postoperatively, CT scan of the abdomen showed a mass at the left bladder wall, 3.7 cm in diameter (Figures 3 and 4). The patient was admitted to our Department of Urology for further evaluation.

The medical history was negative for any urological abnormalities or diseases. The patient did not report any symptoms from the genitourinary system, and the physical examination did not indicate any pathology. Flexible cystoscopy (IMAGE1 S™; Karl Storz, Tuttlingen, Germany) [8, 9] and bladder washout cytology were normal. The patient was submitted to open retropubic surgical exploration for mass removal. The histological examination revealed a benign neoplasm (3.3 × 2.7 × 2.2 cm) composed of dense intersecting fascicles of slender tapered smooth muscle cells, without mitotic activity, nuclear atypia, or areas of necrosis, arranged in a collagenous stroma containing vessels with mural hyalinization. Immunohistochemically, tumor cells expressed muscle antigens such as SMA and desmin, while they were negative for S-100 protein and CD34, [6]. Based on these findings, the diagnosis of BL was set. To the best of our knowledge, this is the first report of a patient presenting with SFT of the pleura and synchronous BL [10].

## 3. Discussion

The first reference for SFTs was made in 1870, and the first complete description of pleural SFTs in 1931 [11]. They are tumors of mesenchymal origin. Differential diagnosis includes various benign and malignant tumors, such as nerve sheath tumors, type A thymomas, sarcomatoid mesothelioma, and synovial sarcoma. Final diagnosis is based on the histopathological findings, corroborated by the immunohistochemical expression profile of the tumor cells. Immunohistochemical markers and their correspondent rate of expression in pleural SFTs are shown in Table 1; with the most specific being STAT6, which is positive in >95% of the cases. Even though the vast majority of lesions are CD34-positive, this is a nonspecific finding. Furthermore, Bcl-2 and CD99-positive staining is nonspecific and not helpful. SFTs are usually benign, although in 10-15% of the cases they may be metastatic or recurrent [12]. According to the World Health Organization, SFTs are classified as intermediate-rare metastatic tumors [5]. The main features of potential malignancy include tumor size (>15 cm), patient age (>55 years), and the presence of mitotic activity > 4/10 high fields of view (×400 magnification) [6]. None of these features was documented in our case, supporting a benign nature of the tumor.

**Figure 5:**
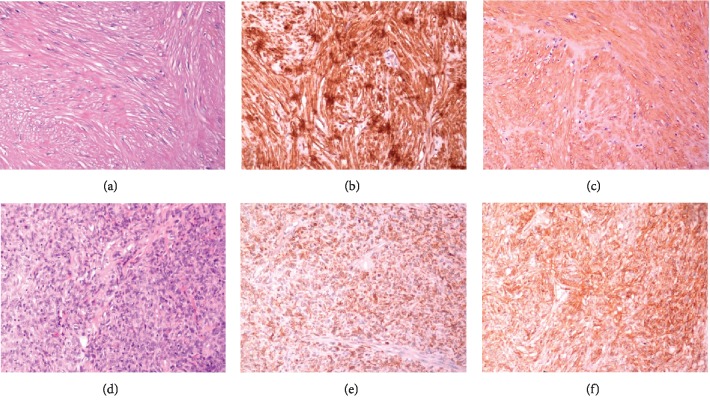
(a–c) Representative images of leiomyoma arising from the muscular wall of urinary bladder stained for H&E (a), desmin (b), and SMA (c), magnification ×200. (d–f) Representative images of pleural solitary fibrous tumor stained for H&E (d), Bcl-2 (e), and CD34 (c), magnification ×200.

BLs are tumors of mesenchymal origin, predominately found in women (76%) [13]. Most patients exhibit symptoms such as obstructive urinary symptoms, including acute urine retention, hematuria, and renal colic, while they may also be asymptomatic [13]. In the present case, BL was an incidental finding. Although patient's SFT of the pleura had no malignant characteristics, the possibility of metastasis had to be ruled out. Cystoscopy and negative cytological examination of bladder washout excluded the presence of an endovesical tumor. Imaging methods such as ultrasound, CT, and magnetic resonance imaging cannot differentiate mesenchymal tumors, and surgical removal of the mass is necessary for definite histopathological diagnosis [14]. The surgical methods that have been applied include laparoscopically assisted, robotically assisted, open, transurethral, and transvaginal excision [15] depending on the tumor location. In this case, the tumor was excluded by an open method since endovesical pathology had been excluded. The final diagnosis (extravesical BL) was based on the histopathological characteristics and the immunohistochemical characterization of the tumor cells. Table 1 and Figure 5 present the immunohistochemical markers and features of SFTs/BLs.

According to Moertel's classification for multiple primary cancers and multicentric cancers, multiple primary cancers are grouped into three groups [16]: group I (multiple primary cancers occurring in organs of the same histology), group II (multiple primary tumors originating from different tissues), and group III (cancers from different tissues/organs concurrently existing with group I cancers, and they form multiple primary cancers of three or more cancers). Group IA includes cancers that occur in the same tissue and organ, group IB includes cancers that are from the same tissue and different organs, and group IC includes cancers that occur in bilateral organs. Multiple primary cancers diagnosed simultaneously or within 6 months are termed as synchronous, while those developing at more than a 6-month interval are termed as metachronous [16]. Based on these classifications and definitions, our case is included in group II, and the tumors are considered synchronous.

The presence of multiple primary tumors may be incidental. Nevertheless, multiple primary tumors can be the result of inherited genetic/immunological defects [17]. To the best of our knowledge, synchronous BLs/SFTs are not related to any known syndrome. Nevertheless, coexistence of two mesenchymal tumors at different sites, in a young patient, may raise the suspicion of a new clinical syndrome that warrants further investigation.

## 4. Conclusion

BLs are very rare benign tumors that possibly coexist with SFTs in extremely rare cases. Surgical exception is the treatment of choice and confirms the diagnosis. Synchronous BL/SFT is not related to any known syndrome and may be incidental. Nevertheless, coexistence of two mesenchymal tumors at different sites, in a young patient, may raise the suspicion of a new clinical syndrome that warrants further investigation.

## Figures and Tables

**Table 1:** Immunohistochemical markers in solitary fibrous tumors (SFTs) of the pleura and bladder leiomyomas (BL) [11, 18].

IHC tumor marker	SFTs of pleura	BL
STAT-6	+ (>95%)	−
CD34	+ (90-100%)	−
Bcl-2	+ (94.3-100%)	Mostly −
Vimentin	+ (100%)	+
Calretinin	− (0-13% +)	−
Desmin	− (0% +)	+
SMA	− (0% +)	+
